# Response of Woodpecker's Head during Pecking Process Simulated by Material Point Method

**DOI:** 10.1371/journal.pone.0122677

**Published:** 2015-04-22

**Authors:** Yuzhe Liu, Xinming Qiu, Xiong Zhang, T. X. Yu

**Affiliations:** 1 Department of Engineering Mechanics, Tsinghua University, Beijing 100084, China; 2 Department of Mechanical and Aerospace Engineering, Hong Kong University of Science and Technology, Clear Water Bay, Kowloon, Hong Kong; 3 Y.K. Pao Chair Professor, Ningbo University, Ningbo, China; Martin Luther University, GERMANY

## Abstract

Prevention of brain injury in woodpeckers under high deceleration during the pecking process has been an intriguing biomechanical problem for a long time. Several studies have provided different explanations, but the function of the hyoid bone, one of the more interesting skeletal features of a woodpecker, still has not been fully explored. This paper studies the relationship between a woodpecker head’s response to impact and the hyoid bone. Based on micro-CT scanning images, the material point method (MPM) is employed to simulate woodpecker’s pecking process. The maximum shear stress in the brainstem (SSS) is adopted as an indicator of brain injury. The motion and deformation of the first cervical vertebra is found to be the main reason of the shear stress of the brain. Our study found that the existence of the hyoid bone reduces the SSS level, enhances the rigidity of the head, and suppresses the oscillation of the endoskeleton after impact. The mechanism is explained by a brief mechanical analysis while the influence of the material properties of the muscle is also discussed.

## Introduction

Woodpeckers drum and drill the trunk of trees to forage for insects hidden under the tree’s surface. According to previous studies, a woodpecker can drum 18 to 22 times per seconds, and 12,000 times a day. During the drumming and drilling process, the peak velocity of a woodpecker’s head is approximately 6–7m/s, and the head deceleration is more than 1200 g on impact [1,2]. This considered, there is no observation of head injury in woodpeckers. In contrast, a human under such high deceleration would sustain significant brain damage.

The structures and constituent parts of woodpecker’ head were investigated in some studies. From the perspective of anatomy, Bock argued the force would be directed away from the dorsal part of the skull towards ventral part of the skull, so the brain is protected [3–5]. The detail description of the drumming process was given by May et al, who claimed the translational trajectory of pecking was the chief reason [6,7]. Oda et al constructed an experiment and the corresponding FEM model, implying that the shape of skull and the hyoid bone served to decrease the stress of brain [8]. By studying the property of materials, Yoon et al illustrated shock-absorbing capability of spongy bone and developed a new bio-inspired shock-absorbing system [2,9]. A concise dimensional analysis given by Gibson exhibited that the small size, the short contacting time and the large contacting area provided the reasons as to the resistance of woodpeckers to head injury [10]. Wang et al showed in a study, consisting of the measurements of the pecking trajectory and the force, an experimental study on skull bones and a FEM simulation, that the longer lower beak reduced the pecking force significantly [1]. Zhu et al carried out numerical studies about the stress wave propagation and the frequency response to impact [11,12]. Nayeon Lee et al studied the beak of the woodpecker experimentally, and found that woodpecker beak had more elongated keratin scales which can dissipate the impact energy by shearing, while at the nanoscale, the beak has a wavy suture which will admit local shearing [13].

**Fig 1:**
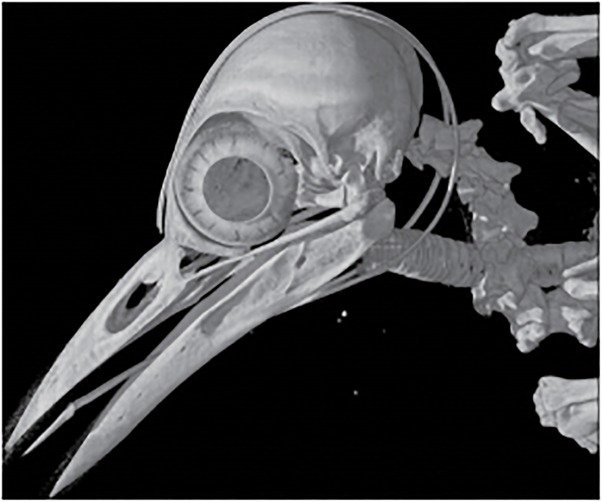
Head structure of *Melanerpes Aurifrons* Golden-fronted Woodpecker. The photo is downloaded from (http://digimorph.org/specimens/Melanerpes_aurifrons/).

To resist the impact, woodpeckers have evolved several special endoskeletal features like a long hyoid bone which encircles the skull, a straight pointed beak unequal in length between the top and bottom beaks, a narrow subdural space, and a smooth skull (Fig 1). Among these distinctions, the hyoid bone is perhaps the most significant. The hyoid bone itself starts at the end of the tongue, passes through the mandible, where it divides into two parts, and then encircles the skull and units into one. It finally ends at the right nostril, which is dissimilar to other birds, where the hyoid bone ends at the mandible. Another fascinating characteristic of a woodpecker’s hyoid bone is that its strength increases from the tip (76MPa) to the root (131MPa), this is comparable with aluminum; and the Young’s modulus of the middle portion (3.72GPa) is more than double of those of two ends, 1.28GPa for the tip and 1.70GPa for the root [14].

However, a reliable quantitative study concerning the function of the hyoid bone has yet to be undertaken. This study shows a delicate material particles method (MPM) model of the endoskeleton, where the muscle and the brain of woodpecker’s head is established based on micro-CT scanning images, and the process of pecking on the trunk is simulated. In order to determine brain damage under the impact, several brain injury criteria are considered, and the shear stress in the midbrain of the brainstem (SSS) was chosen for further investigation. The result substantiated that the special structure of hyoid bone can reduce the risk of impact damage for woodpecker. The analyses about the impact energy show that the hyoid bone and the muscle together will reinforce the structure of woodpecker’s head, thus reducing the deformation and suppressing the oscillation after the separation of the peck from the trunk. The mechanism of this effect of the hyoid bone is explained while the influence of the Young’s modulus of the muscle is discussed.

## Method

### 2.1 Introduction of the material point method (MPM)

In 1994, Sulsky et al proposed the Material Point Method (MPM) [15,16], in which all materials are discretized into a collection of particles carrying mechanical variables e.g., displacement, velocity, strain, stress and kinetic energy. In each time step, particles are rigidly attached to a predefined background grid. After the equations of motion are solved in the grid, results are mapped back to the particles to update their velocities and positions. Then, the deformed background grid is renewed in the next time step.

MPM has proved to be remarkably effective in large deformation, hyper-velocity impact and explosion problems [17–19]. In modeling, the particles can be directly obtained from pixel points of CT scanning images of actual objects. Employing this process, studies of human bone and aluminum foam have been carried out [20–22]. In the present study, the 3D material point method code MPM3D [23] developed by Computational Dynamics Lab in Tsinghua University, is employed in analyzing the dynamic response of a woodpecker’s head during a pecking process.

### 2.2 Simulation model of the pecking of a woodpecker

Using X-ray scanning video provided by the Digital Morphology library at the University of Texas at Austin, the geometrical model of a woodpecker’s head is constructed. The model consists of pixel points with information of xyz coordinate and HU (Hounsfield Unit) value. Then, by selecting the proper HU value range, the endoskeleton and the muscle are specified, and by Boolean operation, the brain is obtained. The lower beak and some vertebrae are rotated and translated to the pose of drumming. The head model is composed of 8 components including brain, forehead, upper beak, lower beak, skull, neck, hyoid bone and muscle. Each component is divided into several segments, and each segment is a collection of pixel points which give the locations and the masses of material particles in MPM calculation. Since the head rotation and brain deformation is not large during the woodpecker pecking, the effect of CSF is omitted in this study, the brain is directly attached to the skull interior face.

Then, the domain which encompassed the whole model is divided into many tiny cubes which have side length *l*_*p*_, or pixel pitch, which statistically represents distance between two nearby points. Therefore, by counting the number of tiny cubes in which at least one pixel point is contained, the volume of each segment is estimated. Then, from the densities listed in Table 1, the total mass of each segment is also calculated. Since the HU value is proportional to the density, it is also proportional to the mass converged on each particle, which can be calculated by

$$M_{i}=\frac{HU_{i}}{\sum_{j=1}^{n_{i}}HU_{j}}\times \left(\rho \cdot n_{t}\cdot l_{p}^{3}\right)$$ (1)

where *M*_*i*_ and *HU*_*i*_ are converged mass and HU value of particle *i*, respectively; *ρ* is density; *n*_*t*_ is the number of tiny cubes occupied; *n*_*i*_ is the number of particles; *l*_*p*_ is the pixel pitch.

**Table 1:** Materials properties adopted.

	Density (kg/m^3^)	Young’s Modulus (GPa)	Poisson ratio
Hyoid Bone	1040[14]	1.28–3.72[14]	0.4[14]
Forehead	1456[8]	0.31[1]	0.4[1]
Upper Beak	1456[8]	1.00[1]	0.3[1]
Lower Beak	1456[8]	1.00[1]	0.3[1]
Neck	1456[8]	0.31[1]	0.4[1]
Skull	1456[8]	0.31[1]	0.4[1]
Brain	1040[22]		
Muscle	1070[22]	1 MPa[22]	0.45[22]
Wood	900[25]	1.49[25]	0.49[25]

The trunk pecked by the woodpecker is represented by a brick composed of particles, whose separation distance is set to be the same as the pixel pitch of the scanning image. The size of the trunk brick is selected to be large enough to avoid the influence of wave reflections. The thickness direction of the trunk is set to be along with the negative Z direction. The simulation model of the woodpecker’s head is rotated in order to make the pecking process a central impact.

Because the contact time of pecking is very brief (found to be in the order of milliseconds [1,10]), and the contact force is relatively large, the gravity and constraint force on the neck side are neglected. During the pecking process, the posture of the neck depends on both the shoulders and the head. Here only the vertebrae near the head are considered to be constituents of the neck, and the boundary on the neck side is assumed to be free. In our study, the calculation duration is 6ms which is short compared to the whole cycle of pecking (about 50–60ms), so the free boundary assumption is acceptable.

In the calculation, because this study is mainly focused on the influence of the woodpecker’s macroscopic structure, the bone is assumed to be homogeneous material. In the MPM model based on the micro-CT scanning image, the mesh size adopted is 0.8mm, which is large enough to neglect the interior structure inside the bone [24]. And when the mesh sizes are 0.9mm and 1.0mm, the variation of the peak of the SSS are about 3% and 7% compared to mesh size of 0.8mm, respectively. So the mesh size adopted is fine enough to get a converged solution.

**Fig 2:**
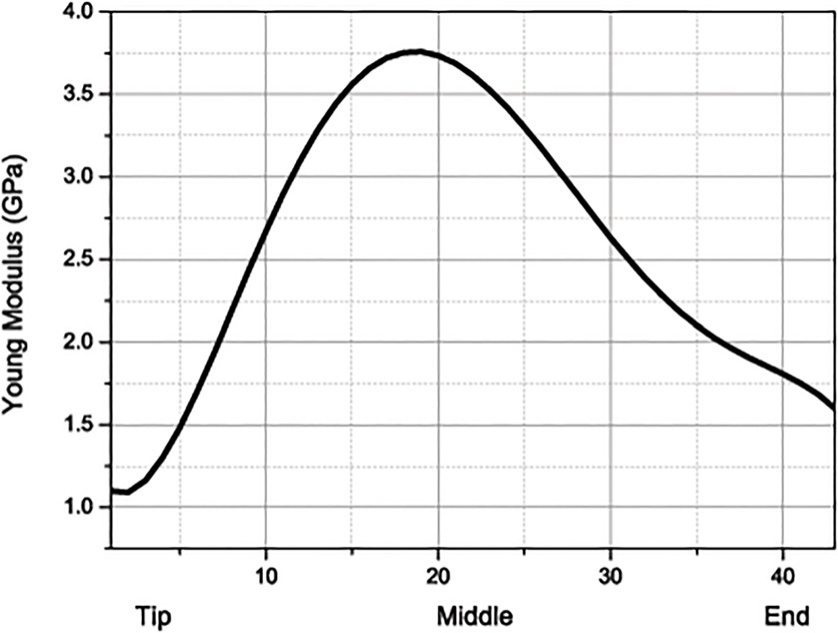
The distribution of Young’s modulus of the hyoid bone.

The beaks, forehead, skull, neck, hyoid bone, muscle and wood are simplified as elastic materials (Table 1). The mechanical property of the wood is estimated from [25]. As mentioned above, the Young’s modulus of the hyoid bone varies from the tip to the end. In the simulation, its distribution is obtained by interpolation, as shown in Fig 2. It has also been shown that the mechanical property of muscle is different at different tension level [26]. However, measuring the muscle’s modulus during pecking has proven very difficult. This being considered, the influence of muscle’s modulus will be investigaed by varying its values in calculations. For the purpose of our study the brain of a woodpecker is assumed to be the same as that of a human, whereby the volume deformation conforms elasticity while the shear deformation conforms viscoelasticity [22]. The shear modulus is given by

$$G\left(t\right)=G_{\infty }+\left(G_{0}-G_{\infty }\right)\cdot e^{-\beta t}$$ (2)

where *G(t)* is the shear modulus at current time; *G*_*∞*_ is the long term shear modulus; *G*_*0*_ is the short term shear modulus and *β* is the decay factor. Here *G*_*0*_ = 0.528MPa, *G*_*∞*_ = 0.168MPa *β* = 35, bulk modulus of 2.19GPa, and Poisson ratio of 0.49, are adopted.

### 2.3 Brain injury criteria

In humans, the brain injury criteria are indicators to evaluate the extent of an injury. The most widely accepted head injury criterion is the Head Injury Criterion (HIC) [27–30]. The value for HIC is calculated by integrating the acceleration-time curve of the centroid. For HIC values less than 1000 a human brain is considered to be safe. It should be pointed out, however, that the rotational movement and the relative movement with respect to the centroid of the brain are not taken into account in the criterion of HIC. Based on the hypothesis that the head injury occurs when the rate of change of kinetic energy exceeds a limiting value, HIP criterion is proposed in [31]. The maximum acceleration (Ar) is also used as a criterion in some circumstances. Using actual field accident data from mild traumatic brain injury cases sustained in American football games, a study which accomplished FE simulations to reconstruct impact events and delineate causation pointed out that the maximum shear stress in the brainstem (SSS) is the best indicator of brain injury [32].

## Results

In order to investigate the role of the hyoid bone in brain damage prevention, two MPM simulation models are established: the original model according to the original structure of woodpecker’s head; and the no-hyoid bone model, in which the effect of hyoid bone is eliminated by setting its modulus to be zero. The brick which represents the trunk is fully fixed on the back surface. Initially, a velocity condition is applied on the full head of woodpecker, which varies from 1m/s to 6m/s. The corresponding values for SSS, HIC, HIP and Ar and the threshold values for humans are given in Table 2. Clearly, due to the difference of size, structure, and material properties, the brain injury criteria of human cannot be applied to woodpecker directly. Although the responses of both models will vary under different impact velocities, the value of impact velocity will not affect the role of the hyoid bone, since most of the materials adopted are elastic except the brain. Here, only the responses under velocity 1m/s are discussed. The true responses under accurate impact velocities will be left for further studies.

**Table 2:** Predictor of brain injury criteria at different impact velocity and the thresholds value for human [25,28,29].

Brain Injury Criteria	1m/s	2m/s	3m/s	4m/s	5m/s	6m/s	human
Maximum SSS (kPa)	2.9	6.2	10.8	15.1	19.7	25.0	6.6
Average SSS(kPa)	1.7	3.8	6.1	9.0	11.4	14.1	
HIC	1107	5969	14913	26933	38580	42741	1000
HIP(W)	5.4	20.9	49.0	79.6	119.2	170.1	1.1×10^4^
Ar(10^3^g)	0.42	0.84	1.32	1.73	2.16	2.60	80g

* The threshold of HIP for human is obtained by assuming the probability of injury is the same as the threshold of SSS, which is 31%.

**Fig 3:**
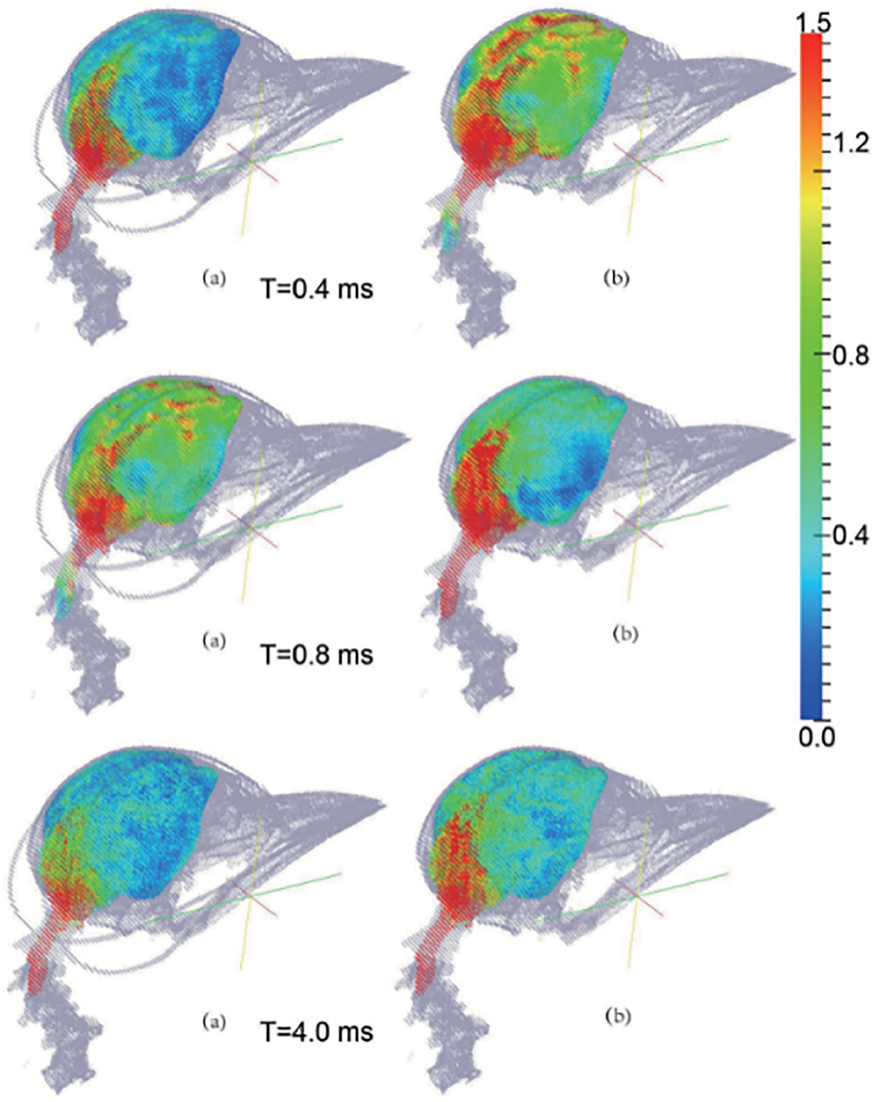
The contours of shear stress of the brain at different time under 1m/s impact velocity. (a) by the original model, and (b) by the no-hyoid bone model. (The unit of the color bar is *KPa*).

**Fig 4:**
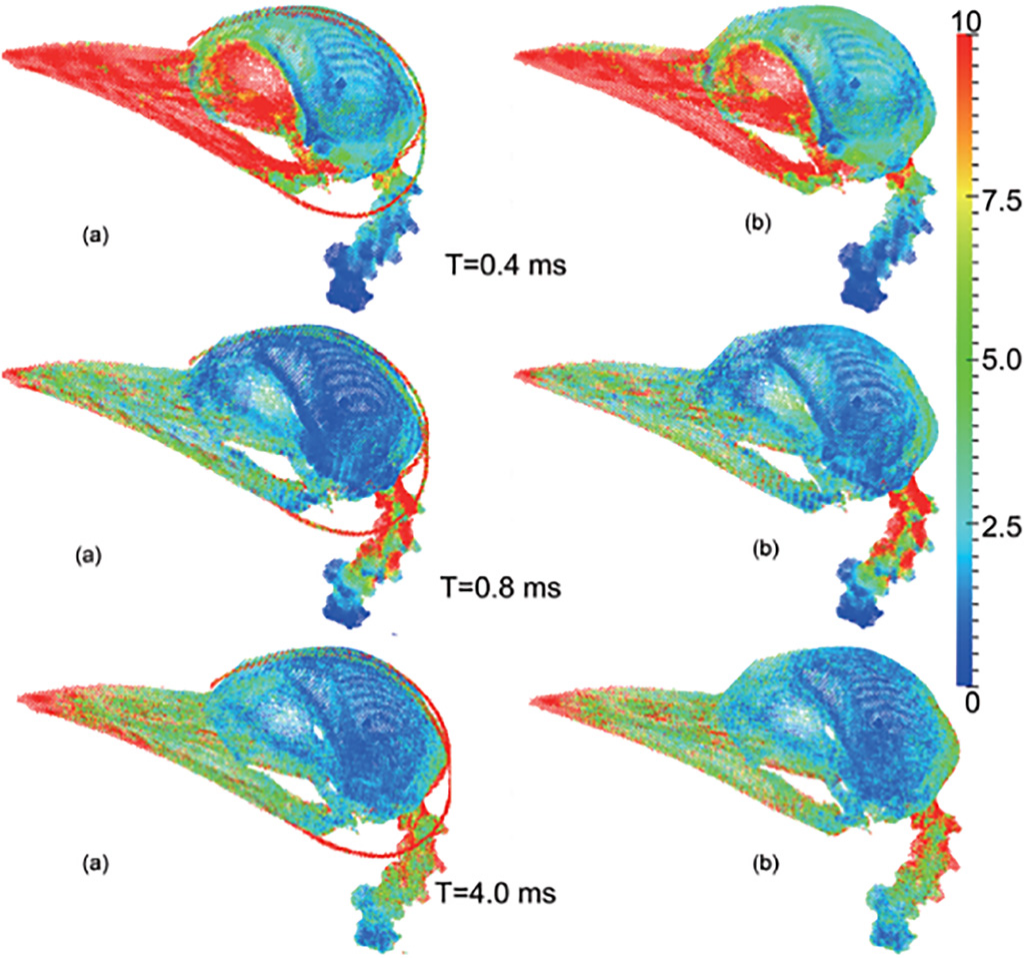
The contours of von Mises stress of the endoskeleton at different time under 1m/s impact velocity. (a) the original model and (b) the no-hyoid model. (The unit of the color bar is 1×10^5^ *Pa*).

The mechanical responses of the brain and the endoskeleton given by these two models are demonstrated in Figs 3 and 4. Contours of the maximum shear stress and the von Mises stress at three different times, 0.4ms, 0.8ms and 4.0ms, are plotted. At instant *t* = 0.4ms, the contact force between woodpecker’s beak and trunk reaches the maximum value; then at *t* = 0.8ms, the beak is separated from the trunk; and at *t* = 4.00ms, the woodpecker’s head has been rebounded for a little while.

As seen from Fig 3, at each time instant, the maximum shear stress given by the no-hyoid model is higher than that by the original model. This implies that the hyoid bone does effectively reduce the risk of the brain damage. This observation is also confirmed by the contours of von Mises stress of the endoskeleton, as shown in Fig 4. From the original model, the hyoid bone is observed in high stress concentration, which is consistent with the result in [12], and the vertebra connected with the skull is found to be in lower stress status than that from the no-hyoid model.

**Fig 5:**
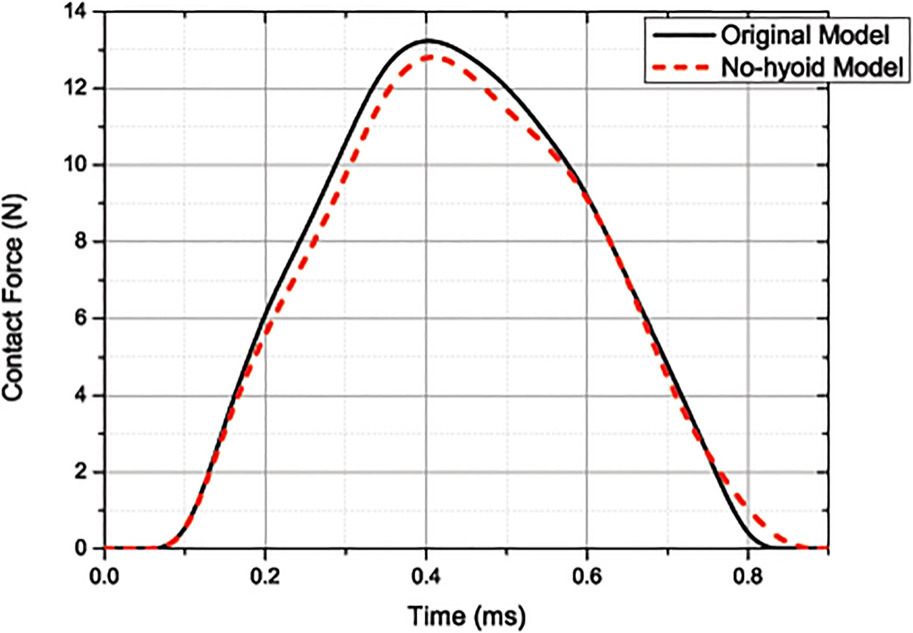
Curves of the contact force between the beak and the trunk for the original model and the no-hyoid model. (under 1m/s impact velocity)

The contact force seen in the two models are compared with each other in Fig 5. The peak value of the contact force in the original model is 13.4N, and the contact time 0.8ms. In the no-hyoid model, the contact force is slightly lower and the contacting time duration is slightly longer; that is, the intensity of the impulse is more slightly moderate. The total impulse is calculated by integrating these two curves, respectively; and they are, 5.71×10^-3^N m for the original model, and 5.49×10^-3^N m for the no-hyoid model.

## Discussions

In this section, the influence of the hyoid bone on the risk of impact damage and the dynamic response of endoskeleton are discussed separately. The influence of the Young’s modulus of the muscle is also considered.

### 4.1 SSS level of the brain after impact

**Fig 6:**
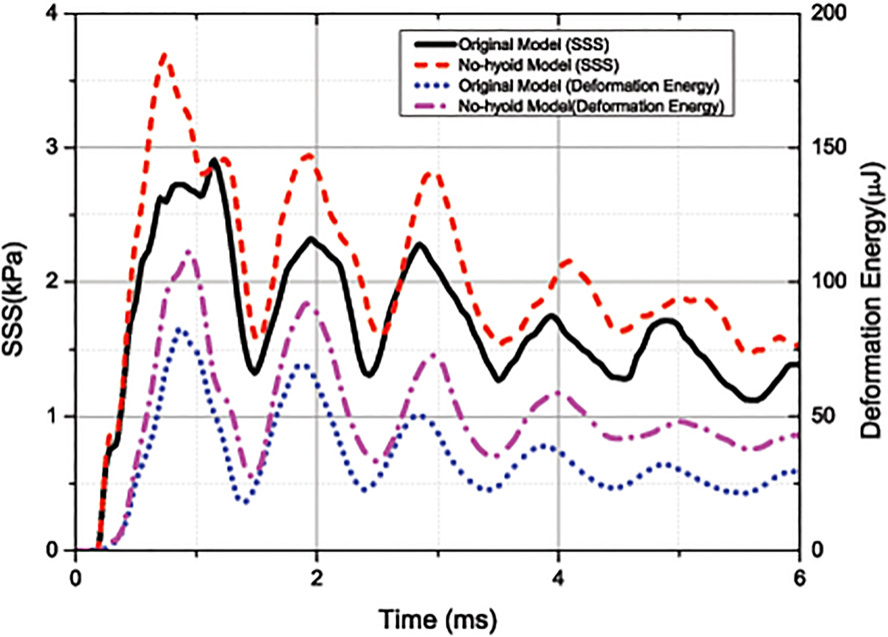
Curves of the average SSS weighted by mass (plotted with the left axis) and the deformation energy of the first cervical vertebra (plotted with the right axis) for the original model and the no-hyoid model. (under 1m/s impact velocity).

The history of the average SSS weighted by mass is shown in Fig 6. The first and highest peak of the SSS curve corresponds to the rebounding instant. The peak and the average value for SSS given by the original models are 2.91kPa and 1.66kPa, respectively; while those given by the no-hyoid model are 3.69kPa and 2.01kPa, which are 26.8% and 21.1% higher than those given by the original model, respectively. Furthermore, the deformation energy of the first cervical vertebra, which connects the neck to the head and is very close to the brainstem, is also shown in Fig 6. The curves of the deformation energy and SSS have the same tendency, and the time they reach the peaks and the valleys are very close.

The similarity of the response of the SSS and the deformation energy can be explained as follows: the high shear stress occurred in the brainstem among the brain is caused by the large deformation of the nearby bone component, i.e., the first cervical vertebra. When the beak hits the trunk, a compressive wave will decelerate the velocities of the skull and brain to zero; then a reflection tensile wave from the back skull will accelerate the head and lead to the rebounding. However, the compressive and tensile waves do not pass by the neck directly, the neck velocity is still forward at the moment of head rebounding. That is, the inertial effect of the neck will cause large relative motion and higher stress in the connection zone. Since the position of brainstem is very close to the first cervical vertebra, and the medulla is even inside the cervical vertebra, the stress of brainstem is sensitive to the relative motion between the skull and neck.

As shown in Fig 1, the hyoid bone bypass the first cervical vertebra; that is, only 2~3mm estimated. And the muscle and other soft tissue provide connection among the hyoid bone, the neck and the skull. Due to the stiffness and position of the hyoid bone, the connection between the neck and head is enhanced; then the deformation and relative rotation of the first cervical vertebra is reduced, so does the peak value of SSS in the brainstem.

### 4.2 Energy distribution during the pecking process

According to the Koenig’s theorem [33], the total kinetic energy is divided into the kinetic energy associated with the global motion of the centroid, $K_{w}^{m}$, and that associated with the local motion relative to the centroid, $K_{w}^{r}$. Considering the deformation energy of the woodpecker head, *E*_*w*_, and the deformation energy of the trunk, *E*_*t*_, the total energy can be written as:

$$E=K_{w}^{m}+K_{w}^{r}+E_{w}+E_{t}$$ (3)

Here *E*_*t*_ is less than 1μJ for both simulation models and thus can be omitted. Clearly, $K_{w}^{m}$ is related to the rigid-body translational motion and will produce neither strain nor stress in the woodpecker’s head. According to the momentum conservation, $K_{w}^{m}$ will remain constant after the woodpecker head is rebounded. *E*_*w*_, which is given by the deformation at a specific time is clearly related to strain and stress. $K_{w}^{r}$, which is determined by the relative motion about the centroid, is converted into a further deformation energy. Thereby, the *relative mechanical energy*, $E_{r}\;=\;E_{w}+K_{w}^{r}$, is defined to characterize the intensity of the impact. From Eq (3), the residual relative mechanical energy *E*_*r*_ should remain constant after the separation of the peck from the trunk. This is because the model does not consider pathway of energy via the neck or the methods to dissipate energy such as viscosity, heat generation in long time. In further study, these factors may be taken into consideration to give more accurate results.

**Fig 7:**
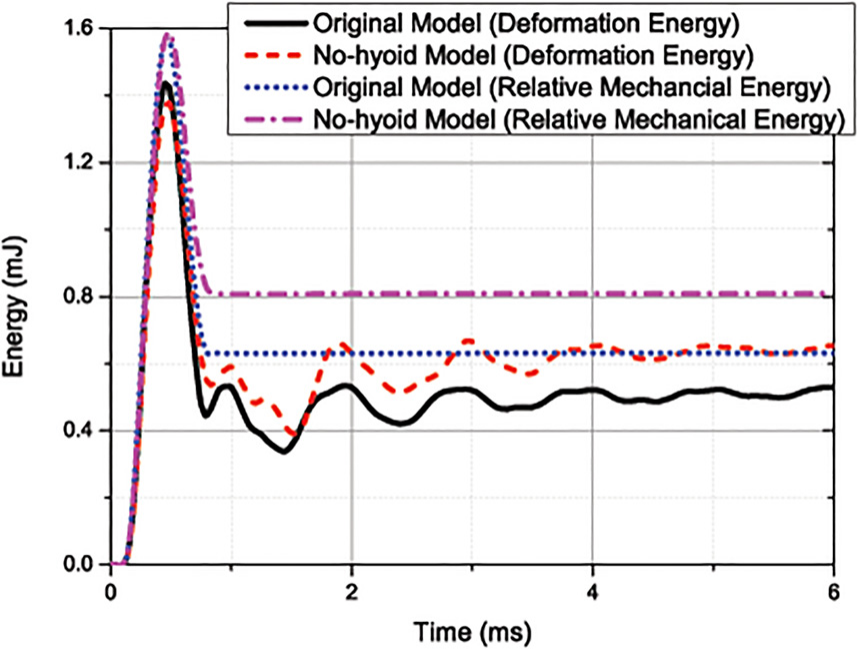
Curves of the relative mechanical energy *E*_*r*_ and the deformation energy *E*_*w*_ of the whole head for the original model and the no-hyoid model. (under 1m/s impact velocity).

Fig 7 shows *E*_*r*_ and *E*_*w*_ as calculated from these two models. Prior to the rebounding of the beak, the curves for *E*_*r*_ and *E*_*w*_ are similar. The only differences occur at the peak. Using the original model, the peak values for *E*_*w*_ and *E*_*r*_ are 1.44mJ and 1.57mJ, respectively. Those by the no-hyoid model are 1.37mJ and 1.58mJ, which are 5.1% lower and 0.6% higher than those calculated using the original model, respectively. After rebound, *E*_*w*_ and $K_{w}^{r}$ is converted into each other during the oscillation, while their total *E*_*r*_ remains unchanged. The value of *E*_*r*_ given by the no-hyoid model is 0.81mJ, which is about 30% higher than that calculated using the original model (0.63mJ). Clearly, the results show that the presence of the hyoid bone results in a slight increase of the maximum deformation during the contact, but at the same time it reduces the severity of oscillation after rebound considerably.

**Fig 8:**
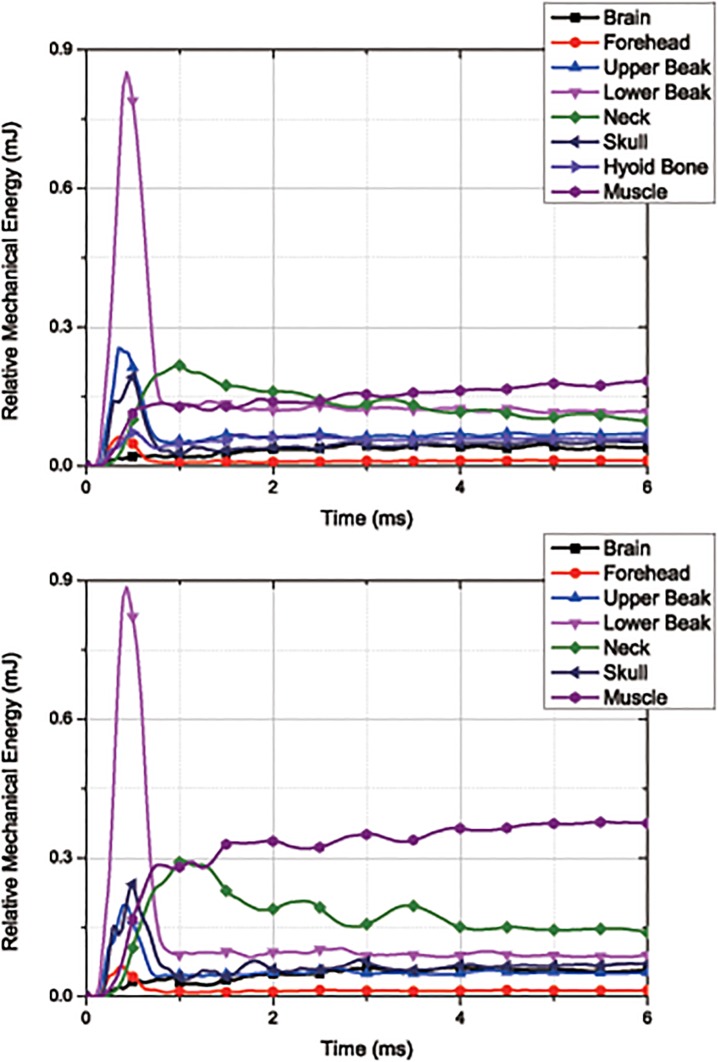
Curves of the relative mechanical energy *E*_*r*_ of different components. (a) the original model and (b) the no-hyoid model. (under 1m/s impact velocity).

In Fig 8, the values of *E*_*r*_ for different components are plotted. Both in the original model and the no-hyoid model, the *E*_*r*_ value of the brain is very low, despite the brain being the heaviest part of woodpecker’s head. Also, since the lower beak is longer than the upper one, its *E*_*r*_ is much higher than those of other parts, which is consistent with the conclusion obtained in [1]. Using the no-hyoid model, the values of *E*_*r*_ for some components are higher than those obtained using the original model, such as the neck and the muscle.

The reason of lower *E*_*r*_ is as follows: the hyoid bone connected to the skull and the forehead is surrounded by muscles and other soft tissue, and it will increase the connection stiffness and reduce the relative motion between the head and neck. Compared with the no-hyoid model, a lower relative mechanical energy is produced even in the cases of higher impulse after rebounding.

In Eq (3), $K_{w}^{m}$ can be calculated by the momentum and the mass of the model, i.e., $K_{w}^{m}\;=\;P^{2}/2m$. And the momentum *P* is equal to the sum of the initial momentum *P*_*0*_ and the impact impulse *I*. As defined above, $K_{w}^{r}+E_{w}\;=\;E_{r}$, whilst *E*_*t*_ is omitted, the Eq (3) can be expressed as:

$$E_{0}=K_{w}^{m}+K_{w}^{r}+E_{w}=\frac{{\left(P_{0}+I\right)}^{2}}{2m}+E_{r}$$ (4)

Since *E*_*0*_, *P*_*0*_, and *m* are identical in the two models, *E*_*r*_ decreases with increasing *I*. As mentioned above, for the original model, the impulse *I* is higher, thus *E*_*r*_ is lower.

### 4.3 The influence of Young’s modulus of muscle and hyoid bone

**Fig 9:**
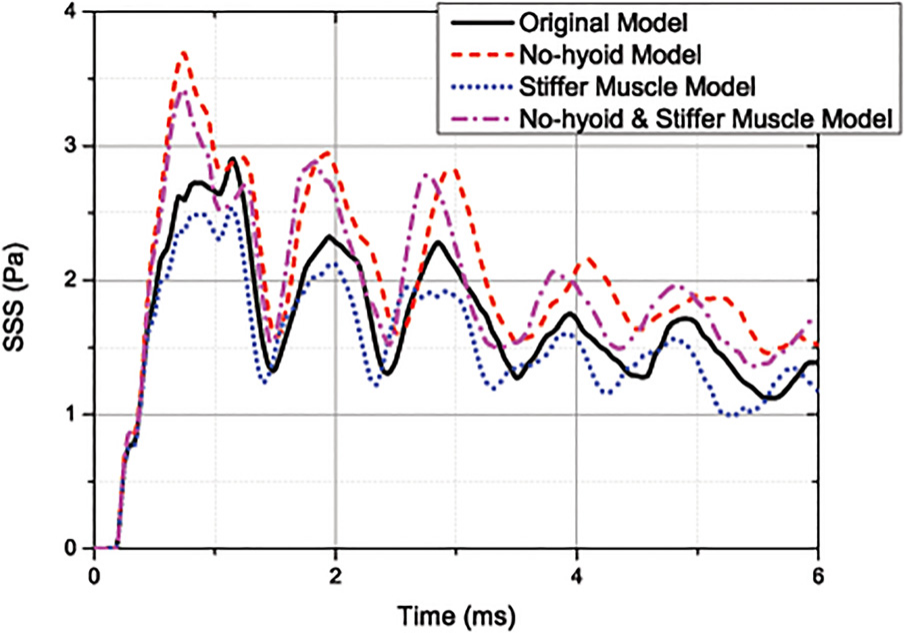
Curves of the average SSS for the original, the no-hyoid, the stiffer muscle and the no-hyoid & stiffer muscle model. (under 1m/s impact velocity).

**Fig 10:**
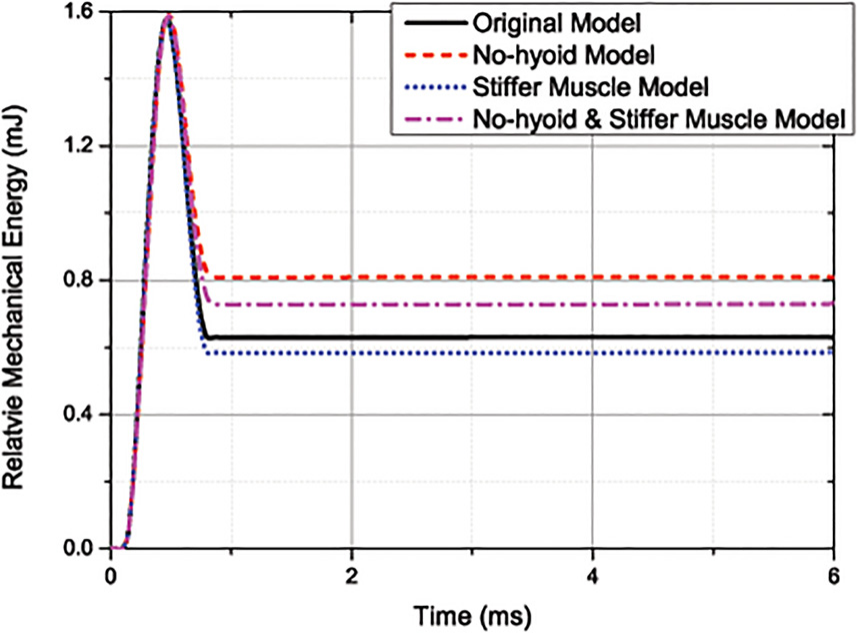
Curves of the relative mechanical energy with time for the original, the no-hyoid, the stiffer muscle and the no-hyoid & stiffer muscle model. (under 1m/s impact velocity).

In this study, the muscle is simplified to be elastic material. When woodpecker pecking, the muscle is controlled precisely to accelerate the head with high efficiency. However, because the physical and morphological background of the muscle’s function is beyond the scope of this study, the muscle is simplified as the passive mass. Then, corresponding to different levels of contraction, the Young’s modulus of muscle is varying in the simulations. Hence, a stiff muscle, which has a Young’s modulus of 1.5 MPa, is used in the calculation, to study its influence on impact response. As shown in Fig 9, when the muscle of the neck is stiffer, the connection between the hyoid bone and other endoskeleton is reinforced. Consequently, the first cervical vertebra is more secure, and the SSS level of brain is further reduced. It should be note that the effect of the stiff muscle itself can also contribute to constraint of the first cervical vertebra. As shown in Table 3, the difference of the first three peaks of the SSS between the original model and the stiffer muscle model is larger than those between the no-hyoid model and the no-hyoid & stiffer muscle model, indicating that the function of muscle on the connection is more important than its function to constrain the neck. In addition, it is found that except the first peak, each peak in the SSS response of the stiffer muscle models happens earlier than the original model and the no-hyoid model. This is attributed to the increase of wave velocity in muscle. It is seen from Fig 10 that the tougher muscle will reduce the residual *E*_*r*_ after rebound, while the peak of *E*_*r*_ remains almost the same. These results are consistent with the above analyses. The function of the muscle was also mentioned in [12], but they did not discuss quantitatively.

**Table 3:** First three peaks for SSS of different models.

Model	1st	2nd	3th
Original	2.91 kPa	2.32 kPa	2.23 kPa
Stiffer Muscle	2.53 kPa	2.12 kPa	1.95 kPa
No-hyoid	3.69 kPa	2.93 kPa	2.81 kPa
No-hyoid & Stiffer Muscle	3.42 kPa	2.87 kPa	2.78 kPa

## Conclusions

By constructing an MPM model of a woodpecker’s head and adopting the maximum shear stress in brainstem (SSS) as a criterion for brain injury, the function of the hyoid bone is studied and is confirmed to reduce the risk of impact damage on the brain to within the limitations of the model. The reason for the reduction is due to the hyoid bone and muscle providing a strong constraint on the first cervical vertebra, thus reducing the relative motion of the neck. The analysis of the relative mechanical energy shows that the hyoid bone and muscle together will enhance the rigidity of woodpecker head, and thus suppress the deformation and oscillation of the endoskeleton after impact. The influence of the Young’s modulus of the muscle is discussed and results are consistent with the conclusion regarding the muscle’s function.

This work reveals some factors by which the anatomy of a woodpecker helps prevent brain injury, and it is hoped that the study will shed light on brain injury prevention in humans. It is also hoped that the function of the hyoid bone may inspire the design of more effective impact protection devices.

## References

[1] Wang L, Cheung JT-M, Pu F, Li D, Zhang M, Fan Y (2011) Why do woodpeckers resist head impact injury: a biomechanical investigation. PloS one 6: e26490 10.1371/journal.pone.0026490 22046293 PMC3202538

[2] Yoon S-H, Park S (2011) A mechanical analysis of woodpecker drumming and its application to shock-absorbing systems. Bioinspiration & Biomimetics 6: 016003. 21245520 10.1088/1748-3182/6/1/016003

[3] Bock WJ (1964) Kinetics of the avian skull. Journal of Morphology 114: 1–41.

[4] Bock WJ (1966) An approach to the functional analysis of bill shape. The Auk 83: 10–51.

[5] Bock WJ (1999) Functional and evolutionary morphology of woodpeckers. Ostrich 70: 23–31.

[6] May PA, Newman P, Fuster J, Hirschman A (1976) Woodpeckers and head injury. The Lancet 307: 454–455. 10.1016/s0140-6736(76)91477-x 55721

[7] May PR, Fuster JM, Haber J, Hirschman A (1979) Woodpecker drilling behavior: An endorsement of the rotational theory of impact brain injury. Archives of Neurology 36: 370 454236 10.1001/archneur.1979.00500420080011

[8] Oda J, Sakamoto J, Sakano K (2006) Mechanical evaluation of the skeletal structure and tissue of the woodpecker and its shock absorbing system. JSME International Journal Series A 49: 390–396.

[9] Yoon S-H, Roh J-E, Kim KL (2009) Woodpecker-inspired shock isolation by microgranular bed. Journal of Physics D: Applied Physics 42: 035501.

[10] Gibson L (2006) Woodpecker pecking: how woodpeckers avoid brain injury. Journal of Zoology 270: 462–465.

[11] Zhu Z, Wu C, Zhang W (2014) Frequency Analysis and Anti-Shock Mechanism of Woodpecker's Head Structure. Journal of Bionic Engineering 11: 282–287.

[12] Zhu ZD, Ma GJ, Wu CW, Chen Z (2012) Numerical study of the impact response of woodpecker's head. AIP Advances 2: 042173.

[13] Lee N, Horstemeyer M, Rhee H, Nabors B, Liao J, Williams LN (2014) Hierarchical multiscale structure–property relationships of the red-bellied woodpecker (Melanerpes carolinus) beak. Journal of The Royal Society Interface 11: 20140274 10.1098/rsif.2014.0274 24812053 PMC4032540

[14] Zhou P, Kong X, Wu C, Chen Z (2009) The novel mechanical property of tongue of a woodpecker. Journal of Bionic Engineering 6: 214–218.

[15] Sulsky D, Chen Z, Schreyer HL (1994) A particle method for history-dependent materials. Computer Methods in Applied Mechanics and Engineering 118: 179–196.

[16] Sulsky D, Zhou S-J, Schreyer HL (1995) Application of a particle-in-cell method to solid mechanics. Computer Physics Communications 87: 236–252.

[17] Ma S, Zhang X, Lian Y, Zhou X (2009) Simulation of high explosive explosion using adaptive material point method. Computer Modeling in Engineering and Sciences (CMES) 39: 101.

[18] Shang M, Xiong Z, Xin-ming Q (2006) Three dimesional material point method for hypervelocity impact [J]. Explosion and Shock Waves 3: 013.

[19] Pan X, Xu A, Zhang G, Zhang P, Zhu J, Ma S, et al (2008) Three-dimensional multi-mesh material point method for solving collision problems. Communications in Theoretical Physics 49: 1129.

[20] Gong W, Liu Y, Zhang X, Ma H (2012) Numerical investigation on dynamical response of aluminum foam subject to hypervelocity impact with material point method. Computer Modeling in Engineering & Sciences(CMES) 83: 527–545.

[21] Gong W, Zhang X, Qiu X (2011) Numerical Study of Dynamic Compression Process of Aluminum Foam with Material Point Method. Computer Modeling in Engineering & Sciences(CMES) 82: 195–213.

[22] Zhou S, Zhang X, Ma H (2013) Numerical simulation of human head impact using the material point method. International Journal of Computational Methods 10.

[23] Ma Z, Zhang X, Huang P (2010) An object-oriented MPM framework for simulation of large deformation and contact of numerous grains. Computer Modeling in Engineering and Sciences (CMES) 55: 61.

[24] Wang L, Zhang H, Fan Y (2011) Comparative study of the mechanical properties, micro-structure, and composition of the cranial and beak bones of the great spotted woodpecker and the lark bird. Science China Life Sciences 54: 1036–1041. 10.1007/s11427-011-4242-2 22173310

[25] Wu J, Yu D, Chan CM, Kim J, Mai YW (2000) Effect of fiber pretreatment condition on the interfacial strength and mechanical properties of wood fiber/PP composites. Journal of applied polymer science 76: 1000–1010.

[26] Hill A (1953) The mechanics of active muscle. Proceedings of the Royal Society of London Series B-Biological Sciences 141: 104–117. 13047276 10.1098/rspb.1953.0027

[27] Gadd CW (1966) Use of a Weighted-Impulse Criterion for Estimatéjlnjury Hazard.

[28] Hutchinson J, Kaiser MJ, Lankarani HM (1998) The head injury criterion (HIC) functional. Applied mathematics and computation 96: 1–16.

[29] Lissner H, Lebow M, Evans F (1960) Experimental studies on the relation between acceleration and intracranial pressure changes in man. Surgery, gynecology & obstetrics 111: 329. 14417481

[30] Versace J (1971) A review of the severity index.

[31] Newman JA, Shewchenko N, Welbourne E (2000) A proposed new biomechanical head injury assessment function-the maximum power index. Stapp car crash journal 44: 215–247. 17458729 10.4271/2000-01-SC16

[32] Zhang L, Yang KH, King AI (2004) A proposed injury threshold for mild traumatic brain injury. TRANSACTIONS-AMERICAN SOCIETY OF MECHANICAL ENGINEERS JOURNAL OF BIOMECHANICAL ENGINEERING 126: 226–236. 10.1115/1.1691446 15179853

[33] Whittaker ET (1952) Analytical dynamics of particles and rigid bodies: University Press.

